# Clinical and Molecular Features of Feline Foamy Virus and Feline Leukemia Virus Co-Infection in Naturally-Infected Cats

**DOI:** 10.3390/v10120702

**Published:** 2018-12-11

**Authors:** Liliane T. F. Cavalcante, Cláudia P. Muniz, Hongwei Jia, Anderson M. Augusto, Fernando Troccoli, Sheila de O. Medeiros, Carlos G. A. Dias, William M. Switzer, Marcelo A. Soares, André F. Santos

**Affiliations:** 1 Departamento de Genética, Universidade Federal do Rio de Janeiro, Rio de Janeiro 21941-590, Brazil; liliane.tavaresdefaria@gmail.com (L.T.F.C.); claudia.muniz16@gmail.com (C.P.M.); sheila.omedeiros@gmail.com (S.d.O.M.); masoares@biologia.ufrj.br (M.A.S.); 2 Programa de Oncovirologia, Instituto Nacional de Câncer, Rio de Janeiro 20231-050, Brazil; 3 Laboratory Branch, Division of HIV/AIDS Prevention, National Center for HIV/AIDS, Hepatitis, STD, and TB Prevention, Centers for Disease Control and Prevention, Atlanta, GA 30329-4027, USA; hjia@cdc.gov (H.J.); bis3@cdc.gov (W.M.S.); 4 Fundação Rio-Zoo, Parque da Quinta da Boa Vista, S/N, Rio de Janeiro 20940-040, Brazil; andersonriozoo@gmail.com (A.M.A.); fernando.troccoli@riozoo.com.br (F.T.); 5 CAT (Centro de Atendimento e Terapia) para Gatos, Rua Mariz e Barros, 292, Rio de Janeiro 20270-001, Brazil; cgabrielvet@hotmail.com

**Keywords:** spumavirus, feline illness, proviral load, neglected virus

## Abstract

Feline foamy virus (FFV) and feline leukemia virus (FeLV) belong to the *Retroviridae* family. While disease has not been reported for FFV infection, FeLV infection can cause anemia and immunosuppression (progressive infection). Co-infection with FFV/FeLV allows evaluation of the pathogenic potential and epidemiology of FFV infection in cats with FeLV pathology. Blood and buccal swab samples from 81 cats were collected in Rio de Janeiro. Plasma was serologically tested for FeLV. DNA extracted from peripheral blood mononuclear cells and buccal swabs was used to PCR detect FFV and FeLV. A qPCR was developed to detect and measure FFV proviral loads (pVLs) in cats. FeLV qPCR was performed using previous methods. The median log10 pVL of FFV mono-infected individuals was lower than found in FFV/FeLV co-infected cats in buccal swabs (*p* = 0.003). We found 78% of cats had detectable buccal FFV DNA in FFV mono-infected and FFV co-infected FeLV-progressive cats, while in FeLV-regressive cats (those without signs of disease) 22% of cats had detectable buccal FFV DNA (*p* = 0.004). Our results suggest that regressive FeLV infection may reduce FFV saliva transmission, the main mode of FV transmission. We did not find evidence of differences in pathogenicity in FFV mono- and -dually infected cats. In summary, we show that FVs may interact with FeLV within the same host. Our study supports the utility of cats naturally co-infected with retroviruses as a model to investigate the impact of FV on immunocompromised mammalian hosts.

## 1. Introduction

Foamy viruses (FV) are complex retroviruses that belong to the *Retroviridae* family and comprise a unique genus within the *Spumaretrovirinae* subfamily that naturally infect many vertebrates, including feline, simian, bovine, and equine [1]. FV infection in humans is invariably associated with zoonotic transmission from other primate species [2,3]. Despite causing a highly cytopathic effect in many cell types in vitro [4], little is known about the pathogenic potential of FV in vivo. A single case-control study reported the presence of hematological abnormalities in humans infected with gorilla simian foamy viruses (SFVs) [5]. Other studies failed to report diseases associated with FV in either natural infection of nonhuman primates or in zoonotic infection of humans with SFVs [6,7,8,9,10,11]. It has been hypothesized that FVs are not able to cause disease in healthy, naturally-infected individuals due to functional immune systems that control virus infection, but these results may be limited by the small numbers of infected persons followed longitudinally [12]. Similarly, an absence of disease association with FVs may reflect an absence of systematic studies following infected individuals, animals or humans for long periods of time since other retroviruses can take decades for disease appearance and disease is not always found in all infected individuals. For example, persons with SFV infection can also be co-infected with HIV and HTLV but longitudinal studies have not been reported on disease outcomes in these persons [8,13,14]. Other viruses, including cytomegalovirus, varicella-zoster and herpes simplex, only rarely cause disease in healthy individuals, but when in co-infection with the human immunodeficiency virus (HIV), can become be activated with pathogenic consequences [15]. Similarly, rhesus macaques naturally infected with SFV and experimentally infected with simian immunodeficiency virus (SIV) progress more frequently to simian acquired immunodeficiency syndrome (SAIDS) compared to SIV-monoinfected macaques [16]. SIV/SFV co-infection in that study was associated with higher SIV viral loads (VLs), lower CD4^+^ T-cell counts and lower survival. 

Domestic cats (*Felis catus*) can be persistently infected with feline spumaretrovirus (the updated name for feline foamy virus—FFV). FFV elicits anti-FFV host immune responses. FFV prevalence in these animals ranges between 30–100% depending on sex, age and geographical region analyzed [17,18,19,20,21]. Upon experimental inoculation, FFV is found in oral mucosa cells 2–3 weeks after infection [22], and is initially detected by qPCR in the blood within the first two weeks of infection [23]. In this same study, two peaks of viremia were observed, at days 20 (80–170 FFU/mL blood) and 155 (332–415 FFU/mL blood). Furthermore, domestic cats are naturally infected by two other retroviruses which cause severe immunodeficiency: the feline immunodeficiency virus (FIV), another complex retrovirus, and feline leukemia virus (FeLV), a simple retrovirus characterized by the absence of accessory genes. Infection of cats with FIV or FeLV allows an evaluation of FFV co-infection in hosts with a compromised immune system and determination of FFV pathogenic potential.

FeLV was discovered decades ago in a group of cats with lymphoma [24], is more pathogenic than FIV, and for many years was responsible for more clinical manifestations than any other etiological agent in cats [25]. FeLV prevalence ranges between 1–3.6% in healthy individuals but between 7.3–12.2% in sick cats [26,27,28,29]. The main transmission route of FeLV is oropharyngeal exposure to mucosal secretions containing the virus [30]. Different infection outcomes have been identified in experimentally infected cats over the years. Regressive infection is defined as a transient antigenemia followed by the establishment of low to moderate proviral loads (pVLs) [31,32]. In contrast, viremic cats are considered to have a progressive infection, with high proviral and plasma RNA VLs [32]. Cats with progressive FeLV infection can develop tumors, anemia and immunosuppression and FeLV has also been associated with hematological disorders, immune-mediated diseases, and other syndromes, including neuropathy and reproductive disorders [25].

FIV was first identified in 1986 [33] in domestic cats in the United States (US). FIV prevalence ranges between 0.9–4.3% in healthy cats and between 7.9–31.3% in sick cats [26,27]. FIV is a feline lentivirus with structure, genome and pathogenesis that parallel those of HIV. Infection occurs primarily through biting [34], but can also occur through vertical transmission [35,36]. Experimentally infected cats undergo an initial acute phase, followed by a chronic or clinically asymptomatic phase, which may persist for over eight years, and a final symptomatic phase, also called feline acquired immunodeficiency syndrome (FAIDS) [37]. In this phase, opportunistic diseases are observed more frequently, such as neoplasia, myelosuppression, glomerulonephritis, neurological diseases and stomatitis. The risk of developing lymphoid malignancy is estimated to be 5 to 6-fold greater in FIV-infected only cats compared with uninfected cats [38].

Several studies have investigated co-infection in domestic cats [29,39,40]. Other studies have investigated the virologic factors involved in retrovirus co-infection [41]. But they mostly focused on FIV/FeLV co-infections. A study by Yamamoto et al. showed that a pre-existent FeLV infection enhanced the expression of FIV in the body and increased the severity of transient primary and chronic secondary stages of FIV infection [42]. Only a single study has evaluated the presence of all three retroviruses in cats [17], but was limited only to prevalence estimates and not their possible interactions or associations with disease. In another study, FeLV and FIV co-infection was examined and showed a greater risk for lymphoid cancers in dually infected cats [38]. The combination of susceptibility to multiple retroviral infections and varied possible disease outcomes makes domestic cats an attractive animal model to study the epidemiology and pathogenesis of co-infection by distinct retroviruses. We examined blood and buccal swab specimens of domestic cats in Brazil for detection and quantification of each feline virus to evaluate their potential association with disease and transmissibility in animals with single or multiple retroviral infections.

## 2. Materials and Methods

### 2.1. Specimen Collection

This project was approved by the Ethics Committee on Animal Use of the Center for Health Sciences at Federal University of Rio de Janeiro (CEUA-CCS) (protocol IBO08-07/16). Blood and buccal swab samples from 81 domestic cats were collected between 2013 and 2014 from three different veterinary clinics in Rio de Janeiro (*n* = 54) or captured within the grounds of Fundação Jardim Zoológico da Cidade do Rio de Janeiro (RIOZOO, *n* = 27), an urban area in the north zone of the city. Cats coming from veterinary clinics were selected for this study once they were suspected of FeLV infection, while cats from RIOZOO were selected randomly, independently whether they looked sick or not.

The cats from clinics were not previously vaccinated for FeLV, while for captured animals we are not sure about their vaccination status although the chances they have been vaccinated are minimal, since Brazilian public policies do not include FeLV vaccination. Upon presentation at the clinics or after capture, the cats were weighed and anesthetized with the administration of 10 g of ketamine or 0.5 μg of xylazine per kilogram of body weight. After anesthesia, 1–3 mL of whole blood was collected from the jugular vein by experienced veterinarians and placed into an EDTA blood tube and stored at room temperature until processing. Buccal swabs were collected from 68 cats by rubbing the upper, bottom and side of the oral cavity with a sterile cotton swab.

Hemogram tests displaying blood cell counts of 35 animals were performed by a veterinarian diagnostic laboratory. Hemogram results were kindly provided by Dr. Carlos Gabriel Dias. Cats with low erythrocytes (below 5 milions/µL), hemoglobin (below 8 g/dL) or hematocrit (below 24%) levels were considered anemic.

Twenty-eight cats were followed three years after the initial collection to determine their life status (alive or dead). For FeLV-infected cats, the time elapsed from infection was estimated based on the diagnosis date at the veterinary clinics. For cats whose infection status was unknown before sampling, the time elapsed from infection was considered using the sampling date.

### 2.2. Demographic and Clinical Variable Description

Cats were classified according to gender, age, outdoor access, household type, neuter status and health status. For age, cats were classified as: (a) kitten, up to 6 months old; (b) young, between 6–12 months of age and (c) adult, above one year of age. For feral cats without a known birthdate, age was estimated by veterinarian evaluation. Household status was classified as: (a) single, cats living alone at home; (b) multicat, cats living with up to 10 cats in the same house; (c) shelter, cats living in a shelter or house with more than 10 cats; and (d) feral, cats that live or have lived in the streets before. Cats classified as single, multicat and shelter were also categorized for outdoor access. Cats were considered sick when any clinical signs, including respiratory tract disease (RTD), progressive weight loss (PWL), ocular secretion (OS), anemia, cachexia, ulcers, hair loss, presence of ectoparasites, tooth loss, prolapses, scabies, pemphigus foliaceous, pyometra, sporotrichosis, diarrhea, neurological symptoms, abortion, kidney disease, stomatitis and neoplasia, or hematological changes were reported. Healthy cats were those without any reported clinical signs at the time of specimen collection.

### 2.3. Specimen Processing and Genomic DNA Extraction

Blood samples were centrifuged at 568× *g* for 10 min to separate plasma from cells. Plasma aliquots were stored at −80 °C for FIV/FeLV serologic testing. Peripheral blood mononuclear cells (PBMCs) were obtained from the cellular fraction by Ficoll-Paque PLUS (GE Healthcare, Waukesha, WI, USA) centrifugation and were stored at −80 °C until genomic DNA (gDNA) extraction. gDNA from PBMC and buccal swabs was extracted using the PureLink^®^ Genomic DNA Mini Kit (Invitrogen, Carlsbad, CA, USA) and was quantified using a NanoVue Plus™ Nanodrop (GE Healthcare). PCR amplification of cytochrome b oxidase gene (*cytB*) sequences was used to verify DNA integrity of the extracted material as previously described [10].

### 2.4. Serological Screening of FIV and FeLV

Plasma from domestic cats was tested for FIV/FeLV by two commercial immunochromatographic tests: the FIV ac/FeLV Ag Test Alere^®^ (Bionote Inc., Gyeonggi-do, Korea) or QuickVET FIV/FeLV Ubio (Biotechnology Systems, Kerala, India) kits at a veterinary clinic and UFRJ, respectively. These tests simultaneously detect an FeLV antigen and an FIV antibody. Assay sensitivity of 100% and 96.7% for FeLV and 96% and 97.6% for FIV, and specificity of 100% and 98.5% for FeLV and 98% and 99.3% for FIV were established by Alere^®^ and Ubio, respectively.

### 2.5. Detection of FFV, FIV and FeLV Using Nested PCR

Nested or semi-nested PCR was performed to diagnose FFV infection by detection of four different FFV genome sequences, including the long terminal repeat (LTR, 215-bp) [43], integrase (*int*, 140-bp) [10], *gag*/polymerase (*pol*) (497-bp) [44] and envelope (*env*, 640-bp) [45]. Three new PCR primers for this study (FFV LTR out, FFV_F1 and FFV_R1) were designed using an alignment of the four available complete FFV genomes from domestic cats (Genbank accession numbers AJ564745, AJ564746, NC001871 and Y08851). Primer sequences are provided in Table 1. Primers used for detection of the FIV reverse transcriptase (RT) portion (603-bp) of the *pol* gene and of FeLV *env* sequences (1689-bp) (Table 1) were published previously ([46]), respectively.

For all published primers, the PCR conditions were followed according to those previous studies [10,43,44,45,46,47]. PCR conditions using newly designed primers were performed using 10× PCR buffer, 25 mM MgCl_2_, 25 mM dNTPs, 25 pmol forward and reverse primers, 1 unit of Taq platinum polymerase (Invitrogen) and 50–200 nanograms of DNA in a total reaction volume of 50 μL. Amplicons from PCR-positive samples were purified using the PCR DNA Mini Kit (Real Genomics RBC, Banqiao, Taiwan) following the manufacturer’s protocol and were sequenced in an ABI 3130xl platform (Thermo Scientific, Waltham, MA, USA).

### 2.6. FFV Quantitative PCR (qPCR)

A new quantitative PCR assay was developed to simultaneously detect and measure FFV proviral loads in infected cats. Generic *pol* primers and probe were designed using an alignment of complete FFV genomes at GenBank. The forward and reverse primers FFVPF1 5′-CAT GTT GTC AGC ACC AAG TAT AC-3′ and FFVPR1 5′-TGC AAG AGA GCA AGT TCT TCT TC-3′, respectively, and probe FFVPFP1 5′-FAM-TTG GAA TTG ATT GTA ATT TAC CAT TTGC- BHQ1–3′ were used to detect a 120-bp *pol* sequence.

Cycling conditions included an initial step of 95 °C for 10 min, followed by 55 cycles of 15 s at 95 °C and 30 s at 60 °C with a final hold step at 4 °C using a Bio-Rad iCycler CFX96 (Bio-Rad Laboratories, Hercules, CA, USA). For assay validation, 20 human blood donor PBMC DNAs and five different human cell lines (MCF-7, CEMx174, Jurkat, LNCaP and HeLa) were used as negative controls to assess assay specificity. Specificity and sensitivity were also determined using PBMC lysates from 39 US domestic cats previously found to be FFV-negative (*n* = 18) or positive (*n* = 21) by Western blot analysis. These cat specimens were available from a previous study investigating the zoonotic potential of feline retroviruses [48]. The limit of detection (LOD) of the assay was determined by testing of 60–140 replicates (depending on the copy number) ten and fivefold serial dilutions of plasmids containing a cloned fragment of the FFV *int* sequence ranging from 100 to a single copy diluted in 1 μg of negative human gDNA. Once the potential LOD was determined using 10 replicates of the 10-fold dilutions, we used a larger number of five-fold plasmid dilution replicates to further refine the LOD. We tested 60 ten copy replicates, 90 five copy replicates and 140 single copy replicates. Standard curves were constructed using 10^0^–10^7^ copies of plasmid DNA. The number of provirus copies/cell was estimated using the formula:

number of copies detected/(DNA quantification in picograms/6 pgs cell)

where the mass for each domestic cat cell genome was considered as six picograms DNA per cell [49]. The amount of DNA in nanograms was measured by spectrophotometry.

### 2.7. FeLV qPCR

The FeLV qPCR test was performed as previously described [49] using a unique region within the U3 of the FeLV LTR with a reported detection limit of 1000 copies per reaction. Specificity of the assay was 100% by obtaining negative results for PBMC DNA from 80 pathogen-free, FeLV-negative cats. Assay sensitivity was also 100% by confirmation of infection in 30 FeLV p27-positive cats. Cats that were experimentally infected and that became persistently antigenaemic as determined by p27 ELISA were considered positive.

The primers and probe FeLV-U3-exo-f 5′-AAC AGC AGA AGT TTC AAG GCC-3′ (forward), FeLV-U3-exo-r 5′-TTA TAG CAG AAA GCG CGC G-3′ (reverse) and FeLV-U3-probe 5′-FAM-CCA GCA GTC TCC AGG CTC CCC A-TAMRA-3′ were used to detect a 131-bp LTR sequence. Standard curves were performed with a plasmid containing the target region in a serial dilution range of 10^3^–10^9^ copies. The cycling conditions consisted of an initial step of GoTaq (Promega, Madison, WI, USA) activation at 95 °C for 2 min followed by 40 cycles of 15 s at 95 °C and 1 min at 60 °C using an ABI 7500 real-time cycler (Applied Biosystems, Foster City, CA, USA). The number of pVL copies/cell was calculated similarly to the FFV pVL, except the pVL was divided by two to compensate for the presence of two LTR copies per integrated provirus.

### 2.8. Classification of FFV and FeLV Infections

We classified FFV-infected cats that were tested for both oral swab and PBMC tissues by nested PCR and/or qPCR. FFV-positive cats without virus detected in saliva were considered as latent infections, and were classified as non-transmissible. FFV-positive cats with virus detected in oral swabs were considered as potential FFV transmitters to other cats and were classified as transmissible.

Cats testing qPCR positive for FeLV were classified into two groups based on VL and serostatus: progressives (those with high pVL, most seropositive) and regressives (those with low VL, most seronegative).

### 2.9. Statistical Analyses 

The Mann-Whitney test was used for statistical comparisons of pVL between groups. Kaplan-Meier survival curves were generated for survival analysis. Linear regression was used to evaluate a correlation between the FFV and FeLV VLs. Odds ratios (OR), 95% confidence intervals (95% CI) and *p*-values were determined to analyze risk factors for FeLV and FFV co-infection, including sex, age, whether neutered, and household status. A multivariate analysis was conducted by adjusting the model for variables that showed significant associations with FFV/FeLV co-infection, i.e., that showed *p*-values below 0.10. To test the different proportion of non- and transmissible cats, probabilities (*p*) were calculated using the Chi-square test. All statistics were performed using MedCalc v.11.3.0.0 or in the *R* environment and *p*-values ≤ 0.05 were considered significant.

## 3. Results

### 3.1. Population Profile

All 81 domestic cats sampled in this study were from Fundação Jardim Zoológico da Cidade do Rio de Janeiro (RIOZOO) and from veterinary clinics throughout the city of Rio de Janeiro. Cats were classified according to gender, age, outdoor access, sexual sterilization, household type, and health status (Table 2). We found an approximately equal proportion of male and female cats and a higher number of adult individuals (57%). Twenty of 36 male cats (56%) were neutered, while 15 of 30 females (50%) were spayed.

Most cats (55%) were classified as feral and of the 35 cats residing in any household (21 multicats, 12 shelters and 2 singles), the majority (69%) had no outdoor access. The majority of cats (66%) were also sick at the time of sample collection. 

### 3.2. FIV and FeLV Infection of Domestic Cats

Blood DNA specimens from all 81 cats were tested by nested PCR and serology for diagnosis of FIV and FeLV infection. Buccal swab genomic DNA (gDNA) specimens available from 68 cats were also analyzed using PCR testing. Animals were considered PCR-positive when at least one viral sequence was detected in PBMCs and/or buccal swab samples. We found three FIV-positive animals, of which 2/81 (2.5%) were positive only by serology and 1/81 (1.2%) was PCR-positive only. Of two serologically FIV-positive animals, one was a house cat and the other was a feral cat. The FIV PCR-positive only cat was feral (Table 3). We found 22/81 (27%) animals reactive in the FeLV p27 antigen test in plasma and an equal number of PCR-positive animals but cats did not always have concordant p27 and PCR results. Specimens from five cats were PCR-positive only and five different cats were FeLV p27-reactive only.

Cats positive using at least one assay (serology or PCR) were classified as infected. Among the household cats (single, multi-cat and shelter) and feral cats, FeLV-positive results in both PCR and serological tests tended to be higher in households, 48% (16/35) compared to feral cats 26% (11/43) (*p* = 0.063; Fisher´s exact test; Table 3). Two cats were dually infected with both FIV and FeLV.

### 3.3. PCR Detection and Quantification of FFV

The 81 PBMC and 68 buccal swab gDNA samples were screened by nested PCR for amplification of four different FFV genomic sequences (Table 4). Overall, we found a FFV PCR prevalence of 46% (37/81). We found more cats with *gag*/*pol* PCR-positive results (27/33, 82% in PBMCs and 13/19, 68% in buccal swab gDNA) compared to other viral targets. Of the 37 FFV-positive animals, 33 had both PBMC and buccal swab gDNA specimens available for nested FFV PCR testing. Twenty-nine animals were PCR-positive (88%) in the PBMC compartment, while only 19 (58%) were positive in the buccal swab (*p* = 0.006). Comparing both tissue compartments, 15 animals were positive in both PBMC and buccal swab, while 14 cats were positive only in PBMC and four were positive only in the buccal swab. No significant differences were found when comparing feral to household cats that were FFV PCR-positive in either the PBMC or the buccal swab compartments (*p* = 0.788 and *p* = 0.247, respectively).

We also developed a new quantitative real-time PCR (qPCR) assay to simultaneously detect and measure FFV proviral load (pVL) in cats. Testing of replicates containing serial dilutions of FFV integrase (*int*)-containing plasmids showed that 90/94 replicates (96%) containing five copies/ug DNA tested positive, whereas 96/141 (68%) replicates containing a single FFV copy were qPCR positive. Hence, we set detection limit of the FFV qPCR assay at five FFV copies/ug gDNA or about 8.33 × 10^−1^ copies/10^6^ cells. FFV *int* sequences were not detected in PBMC gDNA from 20 US human blood donors or in five different human cell lines. The sensitivity of the qPCR assay was also evaluated using PBMC lysates from 39 US domestic cats for which the infection status was previously determined by WB testing [48]. Detection of FFV correlated with the WB and/or nested PCR results in 79.5% (31/39) of the samples. The average and median FFV pVLs were 3.49 × 10^4^ and 1.18 × 10^4^ copies/10^6^ cells, respectively. Two WB-positive but qPCR-negative samples also tested negative for β-actin, indicating possible DNA degradation or PCR inhibitors in those samples. Five of the 16 FFV WB-negative animals had low pVLs (577–1209 copies/10^6^ cells), indicating possible infection during the seroconversion window period or latent infection with a revertant antibody response. One cat with an indeterminate WB result (seroreactivity to a single Gag protein) also tested qPCR negative, suggesting possible nonspecific seroreactivity in this animal.

**Figure 1:**
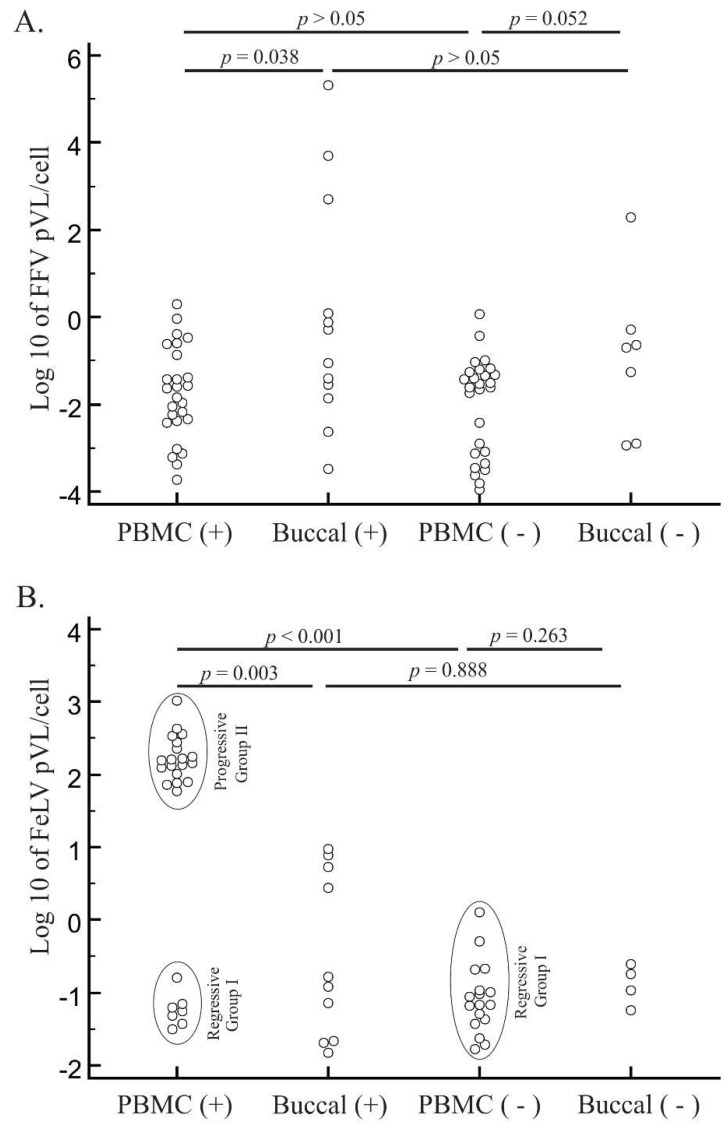
Distribution of feline foamy virus (FFV) and feline leukemia virus (FeLV) log10 proviral loads (pVLs) in blood and oral specimens. FFV (**A**) and FeLV (B) pVLs were measured by quantitative PCR of specimens with nested PCR-positive and -negative test results from domestic cats from Brazil. PCR-positive and -negative cat samples in peripheral blood mononuclear cell (PBMC) or buccal specimens are presented on the *x*-axis. Horizontal lines above each graph depict statistical comparisons between the pVL of specific animal groups or specimen types (Student’s *t* tests) with associated probability (*p*) values shown. Ellipses in (**B**) represent different cat groups of FeLV infection outcomes (regressive or progressive).

PBMC gDNA was further available from 34 of 37 Brazilian cats testing positive using the nested PCR assays. Of these 34 cats, 26 were qPCR-positive with a median pVL of −1.74 log10 copies/cell (18,546 copies/10^6^ cells) and an average of −1.75 log10 copies/cell (175,154 copies/10^6^ cells) (Figure 1A). Of 31 cats with available buccal swab gDNA for qPCR testing, 12 were positive with a median pVL of −0.67 log10 copies/cell (303,671 copies/10^6^ cells). Of the 44 cats testing negative by nested PCR, 39 had PBMC gDNA available for qPCR testing. Of these 39 cats, 27 were qPCR-positive with a median pVL of −1.60 log10 copies/cell (24,900 copies/10^6^ cells). Buccal swab gDNA from 33 nested PCR-negative animals identified seven qPCR positive cats with a median pVL of −0.7 log10 copies/cell (201,363 copies/10^6^ cells). There was no pVL differences between animals that were previously positive or negative for FFV using the nested assays (*p* > 0.05 for both tissues). Interestingly, the pVL was higher in buccal swab than in PBMC specimens (*p* = 0.006). Fifty-three of the 73 PBMC samples tested (73%) were qPCR-positive *versus* 30% (19/64) of buccal swab samples. For FFV-monoinfected cats, we did not find differences between pVL in PBMCs (median pVL of −1.17 log10 copies/cell; *n* = 9) and in buccal swabs (median pVL of −0.98 log10 copies/cell; *n* = 4) (*p* = 1).

By using qPCR, the total number of FFV-positive cats increased from 37 (46%) to 67 (83%). Of these 67 qPCR-positive cats, 26 (38.8%) were monoinfected with FFV. Analysis of cats classified as potentially transmissible and non-transmissible found no statistical difference between FFV pVLs.

### 3.4. FeLV Viral Load Quantification

Of 27 FeLV-positive cats diagnosed by serological and/or molecular assays, 26 with available PBMC gDNA were FeLV qPCR-positive with a median pVL of 2.11 log10 copies/cell (1.29 × 10^8^ copies/10^6^ cells) (Figure 1B). Buccal swab gDNA was available for 13 cats, of which 10 were FeLV qPCR-positive with a median pVL of −0.55 log10 copies/cell (2.88 × 10^5^ copies/10^6^ cells). The FeLV pVL was approximately 1000× higher in PBMC than in buccal specimens (*p* = 0.0081). Our pVL data are correlated with FeLV outcome (progressives of Group I, those cats with high VL, mostly seropositive) and regressives (Group II, those with low VL, mostly seronegative). Group I (*n* = 19) had a higher pVL with an average of 2.23 log10 FeLV copies/cell (2.25 × 10^8^ copies/10^6^ cells) and median pVL of 2.20 FeLV log10 copies/cell (1.59 × 10^8^ copies/10^6^ cells) whereas Group II (*n* = 7) cats had a lower median pVL of −1.25 FeLV log10 copies/cell (5.59 × 10^4^ copies/10^6^ cells) (*p* < 0.001). In this case, there was no difference between serological status of the two groups: in group I, 16% (*n* = 3) were FeLV-seronegatives while in group II, 28% (*n* = 2) had that status (*p* = 0.588).

We also tested the 52 of the 54 cats with negative FeLV serology and/or molecular assays with available PBMC gDNA. We found 17 FeLV-positive cats by qPCR with a median of −1.05 log10 copies/cell (8.9 × 10^4^ copies/10^6^ cells). This median pVL is almost 2000× lower than the median PBMC pVL in FeLV-seropositive cats (*p* < 0.001). pVLs of FeLV-seronegative cats were similar to those measured in the group II FeLV-positive cats (*p* = 0.193), indicating that the FeLV-seronegative cats with low pVLs likely had a regressive infection. Testing of 35 buccal swab gDNA samples identified four qPCR-positive cats with a median FeLV pVL of −0.55 log10 copies/cell (2.91 × 10^5^ copies/10^6^ cells). However, there was no difference between buccal swab pVLs in FeLV-seronegative and seropositive cats (*p* = 0.945). Cats with progressive and regressive infections differed in the qPCR detection of FeLV in buccal tissues. Of 21 regressive animals, only five (24%) had detectable pVL in buccal cells, while all 8 (100%) of animals with progressive infection had detectable pVL in that compartment (*p* < 0.001). Nonetheless, buccal swab pVLs of progressive and regressive animals were similar (*p* = 0.608).

By using qPCR, the total number of FeLV-positive cats increased from 27 (33%) to 48 (59%). Of the 48 qPCR-positive cats, eight (17%) were monoinfected with FeLV, while 38 (79%) were co-infected with both FFV and FeLV and two (4%) were infected with all three feline retroviruses.

### 3.5. Characteristics of Cats with Feline Retrovirus Co-Infection

We found 32% (26/81) of the cats in our study to be FFV-monoinfected, 10% (8/81) with FeLV monoinfection, 47% (38/81) with dual FFV/FeLV co-infection, 1% (1/81) with dual FFV/FIV co-infection, 2% (2/81) with triple FFV/FeLV/FIV co-infection and 7% (6/81) with no evidence of any feline retrovirus. No FIV monoinfections or dual FeLV/FIV co-infections were identified. Therefore, subsequent statistical analyses were based on the three groups consisting of FFV and FeLV monoinfections and FFV/FeLV dual infections, since these infections were more common in our population.

We found an approximate 1:1 ratio between females and males in all three FFV and FeLV infection groups (Table 2). Kittens, young, and adult cats were present in all three groups, except for the FeLV-monoinfected group which only contained adults. The majority of cats living in multi-cat homes or shelters were FFV/FeLV-co-infected (52% and 75%, respectively). The majority of cats with outdoor access and poor health were also co-infected with FFV and FeLV (Table 2), but these differences were not statistically significant. In captive animals, monoinfection of FFV was 11.4% (4/35) while in feral animals the prevalence was 49% (21/43) (*p* < 0.001). However, the total prevalence of FFV (mono and dual-infection with FeLV) was 86% in captive animals and 71% in feral animals (*p* = 0.111). FeLV monoinfection prevalence was lower in feral (5%) compared to captive (14%) cats, and similarly the total prevalence of FeLV was lower in feral cats (42%) than in the captive cats (74%) (*p* = 0.004).

Odds ratios were determined to evaluate whether FFV infection was a risk factor for FeLV infection. Infection with FFV did not increase the chances of having FeLV infection (OR = 1.09, *p* = 0.878). Among FFV-infected cats, odds ratios were also calculated to evaluate risk factors involved with FeLV co-infection (Table 5). Neutered FFV-infected cats were more likely to be co-infected than non-neutered counterparts. Cats living in shelters showed higher rates of co-infection than those cats in other housing types. Non-feral cats living alone, in a shelter or multicat household had a higher risk of co-infection than feral cats (OR = 6.89, *p* = 0.003). No associations of co-infection were found with either age or sex. We also evaluated risk factors for FFV co-infection of FeLV-infected cats. No significant differences were observed when considering the same factors analyzed for FFV-infected cats (OR = 0.9, *p* = 0.878).

Since the street and neuter variables showed significance at the univariate level, a multivariate analysis was performed with adjustment for those two variables. While the effect of neutering was lost (OR = 2.43; *p* = 0.161), street cats remained significantly less prone for co-infection with FFV and FeLV than non-street cats (OR = 0.22; *p* = 0.026).

**Figure 2:**
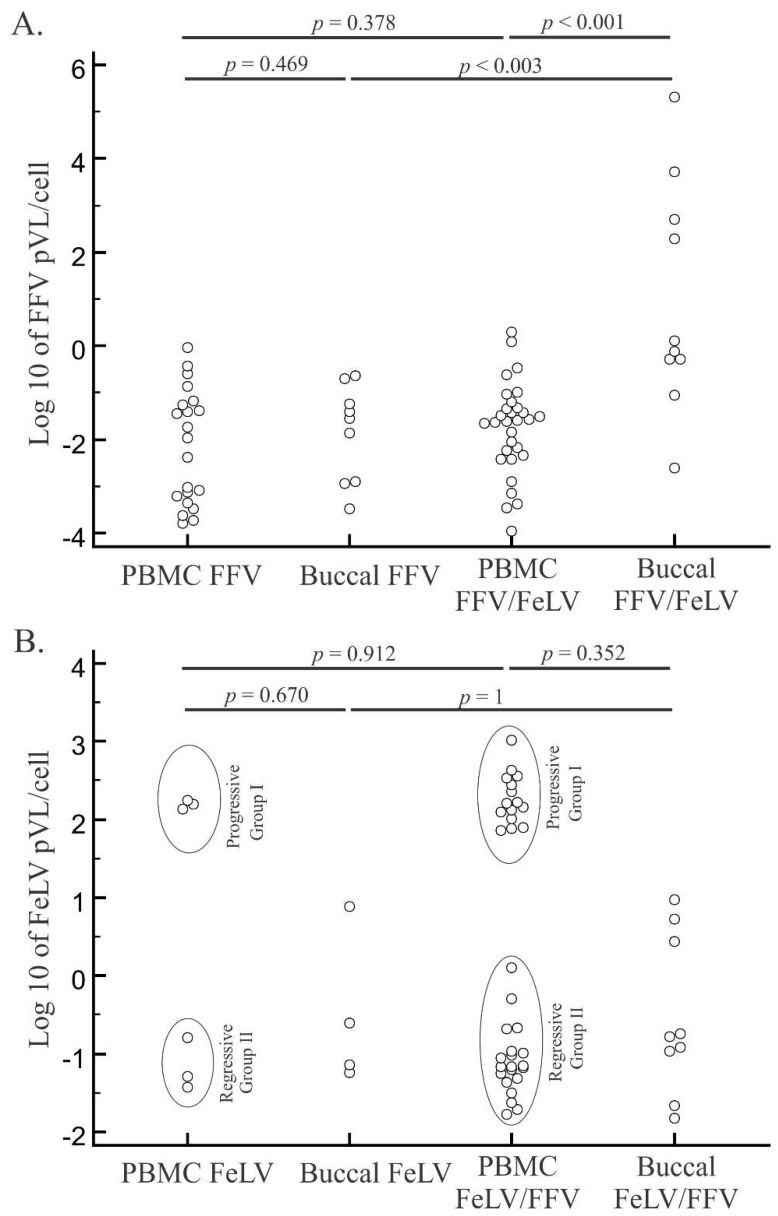
Distribution of feline foamy virus (FFV) and feline leukemia virus (FeLV) log10 proviral loads (pVLs) in blood and oral specimens from singly or dually infected cats. FFV (**A**) and FeLV (**B**) pVLs were measured by quantitative PCR of mono- and co-infected domestic cats from Brazil. Mono- and co-infected cat samples for each retrovirus and in peripheral blood mononuclear cell (PBMC) or buccal specimens are presented. Horizontal lines above each graph depict statistical comparisons between the pVL of specific animal groups or specimen types (Student’s *t* tests), with associated probability (*p*) values shown. Ellipses in (**B**) represent different groups of FeLV infection outcome (regressive or progressive).

When we compared median log10 pVLs of FFV mono- and FFV/FeLV co-infected individuals (Figure 2) we found higher FFV pVLs in buccal swab specimens in co-infected cats (*p* < 0.003; Figure 2A). In contrast, differences in PBMC pVLs between mono- and co-infected cats were not significant (*p* = 0.378). However, pVLs in the buccal specimens were significantly higher than those in PBMC of co-infected cats (*p* < 0.001). No differences were found in FeLV pVLs between buccal swabs and PBMCs or between FeLV mono- and co-infected cats (*p* = 1 and *p* = 0.912, respectively; Figure 2B).

**Figure 3:**
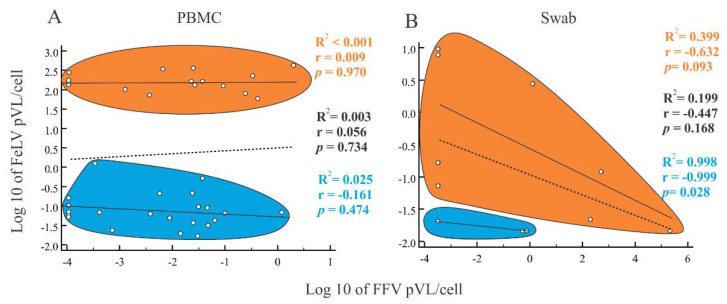
Spearman correlation analysis between feline leukemia virus (FeLV) and feline foamy virus (FFV) log10 proviral loads (pVLs) on blood and oral specimens. Correlations were tested in both peripheral blood mononuclear cells (PBMCs) (**A**) and buccal swab specimens (**B**). Vertical and horizontal axes represent FeLV and FFV pVLs, respectively. Each open circle represents a sample for which pVL was measured for both viruses. Orange and blue ellipsoids represent FeLV progressive and regressive individuals, respectively. Correlation coefficients (R^2^) and probabilities (*p*-values) are provided for each group (progressive and regressive) and for all samples together. They are represented in the graph by lines. Dotted lines represent the correlation among all samples, both progressive and regressive. For PBMCs, progressive and regressive cats had FFV and FeLV pVLs with weak correlation (R^2^ < 0.001, *p* = 0.970 and R^2^ = 0.025, *p* = 0.474, respectively). pVLs in buccal swab samples of progressive and regressive cats were inversely correlated (R^2^ = 0.399, *p* = 0.093 and R^2^ = 0.998, *p* = 0.028, respectively).

We also evaluated pVLs of cat samples that were tested for both FFV and FeLV, but did not observe a correlation between FeLV and FFV pVLs in PBMCs among cats with progressive or regressive infection (Figure 3A). Similarly, there was no correlation between the FFV and FeLV pVLs in buccal specimens (Figure 3B) except for FeLV-regressive individuals (r^2^ = 0.998; *p* = 0.028; Figure 3B), which showed a negative association.

**Figure 4:**
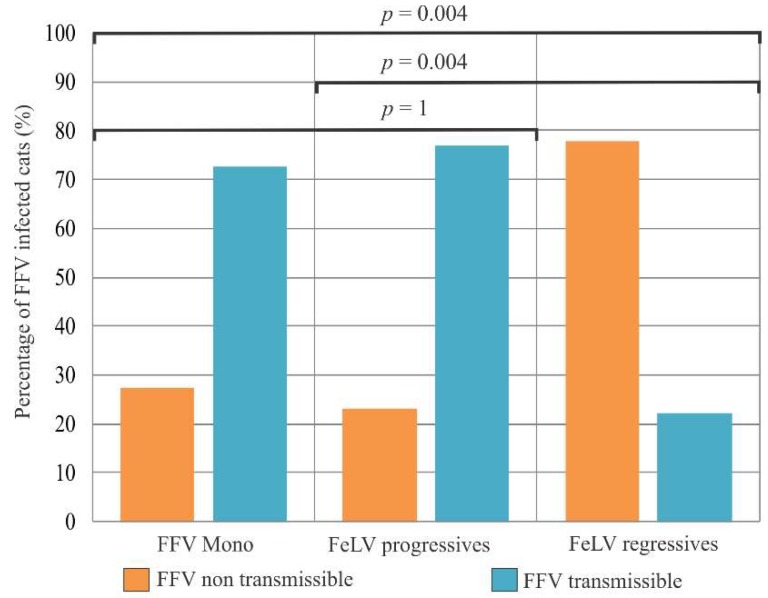
Proportion of potentially transmissible and nontransmisible cats based on proviral loads (pVLs) in different groups of feline foamy virus (FFV). The groups compared include monoinfected cats (FFV Mono), feline leukemia virus (FeLV) progressive (FeLV progressives) and regressive (FeLV regressives) cats in dual infection. In orange, the potentially FFV non-transmissible cats (undetectable buccal swab DNA). In blue, the FFV potentially transmissible cats (detectable DNA). Probabilities (*p*) were calculated using the Chi-square test.

The percentage of potentially transmissible FFV with detectable pVLs in buccal swabs in monoinfected cats (72.7%) was very similar to that in FeLV progressive cats (76.9%) (Figure 4). The opposite was found when comparing FFV-monoinfected cats and FeLV regressives (*p* = 0.004).

For cats with FFV and FeLV positive results for both buccal swab and PBMC specimens, we compared the FFV pVLs between FFV-monoinfected and FFV/FeLV-co-infected animals and also the pVLs between FeLV progressive and regressive animals. However, we did not observe a correlation between pVLs in buccal swab or PBMC in either situation (data not shown).

### 3.6. Association of FFV and FeLV Infection with Clinical Signs

To investigate possible disease associations in cats with mono or dual FFV and FeLV infection we analyzed clinical signs reported in these animals at the time of sampling. We did not detect any differences among the proportions of major clinical signs (RTD, PWL and OS) in FFV-monoinfected, or FeLV-monoinfected regressive or progressive cats (Table 6). Five of seven (71%) cats with FFV/FeLV-progressive infection were anemic at the time of data collection by hemogram testing while none of the FFV/FeLV-regressive cats were anemic (0/2; *p* = 0.026). Less common clinical signs detected were cachexia (2/81), ulcers (2/81), hair loss (3/81), presence of ectoparasites (1/81), tooth loss (1/81), prolapses (1/81), scabies (1/81), pemphigus foliaceous (1/81), pyometra (1/81), sporotrichosis (4/81), diarrhea (1/81), neurological (1/81), abortion (1/81), kidney disease (1/81), stomatitis (3/81) and neoplasia (1/81). However, the low frequency of these clinical signs did not permit an analysis of their association with retrovirus infection of these cats.

We analyzed FFV pVLs in cats with or without each reported major clinical signs, but did not observe differences between pVLs in cats with or without PWL (*p* = 0.456), RTD (*p* = 0.629) or OS (*p* = 0.174). We also did not find any FFV pVL differences in cats with or without any clinical signs (*p* = 0.941), including anemia and death (*p* = 0.885 and *p* = 0.558, respectively).

For FeLV-positive cats (either by serology or PCR), anemia at the time of sampling was correlated with higher PBMC pVL, with a median of 2.29 log10 FeLV copies/cell (197,784,339 copies/10^6^ cells) compared to −1.31 log10 FeLV copies/cell (48,977 copies/10^6^ cells) in cats without anemia (*p* = 0.045, Mann-Whitney *U* test). Death was also correlated with higher PBMC pVL, with a median of 2.20 log10 FeLV copies/cell (161,171,526 copies/10^6^ cells) among cats who died during follow-up compared to a median of −1 log10 FeLV copies/cell (100,000 copies/10^6^ cells) at sampling time among those who were still alive at the end of follow-up (*p* = 0.022, Mann-Whitney *U* test).

**Figure 5:**
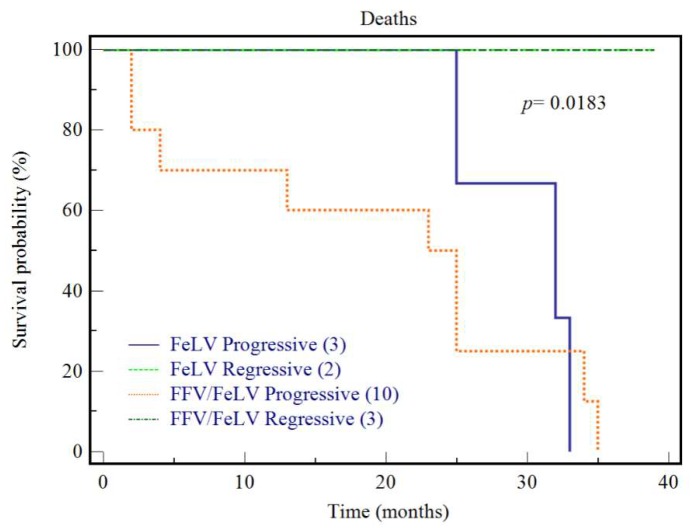
Survival analysis of cats with feline leukemia virus (FeLV) progressive and regressive infections irrespective of their FFV infection status. Kaplan-Meier survival analysis was conducted for cats that were followed-up over 35 months during the study. *p*-value of the comparison (*p*) is shown. Cats with regressive infections were more likely to survive during this period compared to cats with progressive infection (*p* = 0.008).

We followed 21 cats from entry into the study until 35 months later. Among those, three were FFV-monoinfected, and 13 were FeLV progressives and five were FeLV regressives, irrespective of their FFV infection status. After 25 months of follow-up, all three FFV monoinfected cats were alive. After 35 months, 92.3% (12/13) of cats with progressive infection had died (Figure 5), of which three were feral (25%) and nine were captive (75%), 11 were adults (92%) and one was young (8%) whereas the 13th cat was lost to follow-up at 24 months. In contrast, all regressive cats were alive after 35 months (*p* = 0.0008). Among regressive cats, two were feral (40%) and three were captive (60%), while four were adults (80%) and one was a kitten (20%). There was no difference in age or household status in these cat groups. Similar rates of FFV infection were found in both groups; 10/13 (76.9%) in the first group and 5/7 (71.4%) in the second.

## 4. Discussion

In this study, we evaluated FV infection in cats co-infected with other retroviruses known to impair the feline host immune system with pathologic consequences. Retroviral infection of domestic cats was used as a model to investigate possible outcomes associated with mono or dual infections because biological materials such as blood and buccal swabs are usually collected from domestic cats at veterinary clinics for periodic clinical examination and pets can also be easily followed. Moreover, domestic cats can be infected with FeLV, a retrovirus historically considered to be the causative agent of more clinical syndromes than any other single microbial agent in cats, affording an assessment of dual retroviral infections on disease outcomes in cats [25]. Unfortunately, the low FIV prevalence in our study population did not permit an evaluation of dual or triple infection with that retrovirus.

In this study we found that both FFV and FeLV qPCR testing were more sensitive than their respective nested PCR assays for detecting FFV and FeLV, respectively. Even though the FeLV and FFV nested PCR assays in our study targeted multiple viral regions, none were able to detect 100% of the qPCR-positive samples. These results likely reflect viral sequence variation at the primer locations and the target viral genomic fragment size for which detection of smaller amplicon sizes, as those in the qPCR tests, is typically more sensitive. We have obtained similar results when comparing nested PCR and qPCR testing of New World primates (NWP) [9].

In this study, about one fourth of FFV-infected cats were PCR-positive in only the PBMC compartment. These animals may represent recent infections, in which the virus has not yet disseminated to the oral tissues. This hypothesis is supported by a previous report showing a delay in the appearance of SFV in the saliva of cynomolgus macaques (*Macaca fascicularis*) infected through blood transfusion from an SFV-infected animal [50]. Epstein-Barr virus (EBV) shares some features with FV, including a high incidence of infection, a salivary mode of transmission and replication in the oropharyngeal tissues. As it is well established for EBV, it is possible that migratory cells, such as macrophages and other leukocytes initially infected by FV, eventually traffic to the oropharyngeal tissues and spread infection to less differentiated epithelial cells [51].

A recent study with NWP showed an average of −3.1 log10 SFV copies/cell in PBMC [52] whereas in Old World Primates (OWP) the pVL average was −3.08 log10 SFV copies/cell among rhesus macaques [53]. We found a 10× higher FFV pVL (−2.15 log10 copies/cell) in the same tissue compartment in cats. At oral sites, the average NWP SFV pVL was −1.3 log10 copies/cell [52] while in OWPs the pVL ranged from −3.3 to −1 log10 SFV DNA copies/cell [54]. We found similar FFV pVLs in the cat oral cavity (−1.86 log10 copies/cell). Unlike the previous work with primates [52], we did not find significant differences between the pVL in PBMC and in oral tissues of cats. These data suggest that FV pVL distribution at distinct anatomical sites can vary in different natural hosts. We also showed that buccal swabs can be useful as non-invasive biological materials for FFV diagnosis, as recently shown for SFV [52].

Non-feral cats were almost five times more likely to be co-infected with FFV/FeLV than feral cats. We hypothesize that being confined in a household or shelter environment increases the risk of co-infection since these cats may have more intimate contact during grooming or sharing feeding bowls. We also considered possible recruitment bias since clinic cats were suspected to be ill as explained in Methods. These cat behaviors are reported as the most likely routes of FeLV transmission [55], as opposed to biting, which has been more strongly associated with SFV transmission [56]. In our study the FFV prevalence was not different between captive and feral adult cats, while in SFV studies of OWPs the prevalence is higher in adult captive (~70%) [57] than in wild-born primates (~44%) [58], whereas the SFV prevalence in NWP were found very similar in captive and wild animals (14–30%) [10]. These differences can likely be explained by some captive cats having outdoor access and in shelters it is not uncommon for the introduction of new individuals from the street, both increasing the likelihood of exposures to potentially infected cats.

Using qPCR, we identified two distinct cat groups based on FeLV pVL; a regressive group with low pVLs (−1.77 to 0.10 log10 copies/cell) and a progressive group with higher pVLs (1.77 to 3.02 log10 copies/cell). Our results support previous studies showing that pVLs measured by qPCR can be used to identify a regressive infection outcome in which plasma viremia has been cleared [59]. Moreover, the FeLV pVL we detected is within the range reported by others for experimentally and naturally infected cats [60]. However, we show for the first time that naturally-infected cats with progressive FeLV infection can result in death after 35 months of follow-up, although the cause of death cannot be exclusively attributed to FeLV infection. Other studies showed the same outcomes only for experimentally infected cats [60,61]. In one of those studies, progressive and regressive cats were followed for 149 weeks post transfusion and the authors found that 50% (4/8) of regressive cats remained healthy while 100% (2/2) of progressive cats developed non-regenerative anemia and multicentric T-cell lymphoma [61].

**Figure 6:**
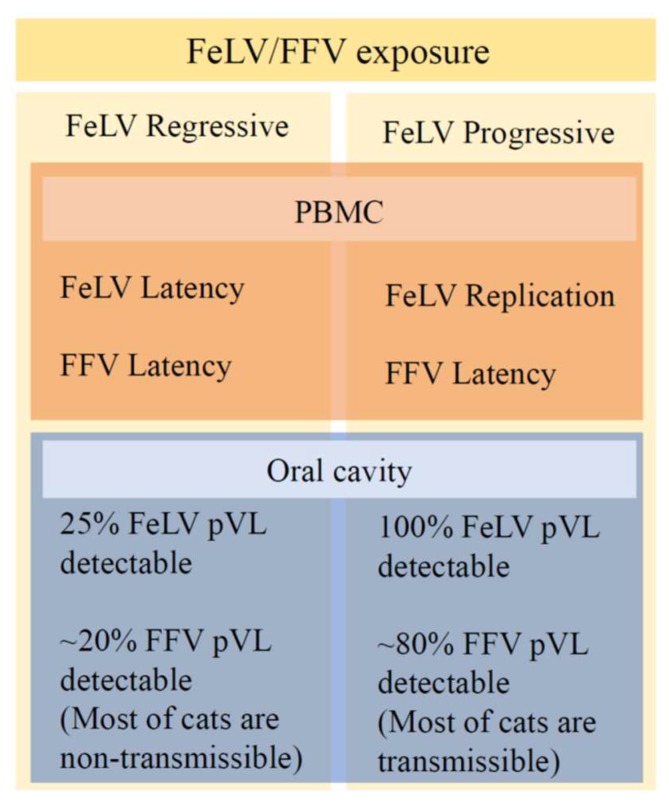
Hypothetical pathway to progressive or regressive outcomes via dual infection with feline foamy virus (FFV) and feline leukaemia virus (FeLV) and potentially active viral replication measured by proviral load (pVL). FeLV progressive and regressive outcome determined by PBMC tissue (blood) pVLs were correlated to different FFV and FeLV pVL patterns of detection in the oral cavity.

To the best of our knowledge, our study is the first to observe the influence of another retrovirus on FV pVL in buccal swabs. A previous co-infection study conducted in the rhesus macaque model has shown that a pre-existing SFV infection can influence the biology and the outcomes of an SIV infection, including higher plasma SIV VL, a decreasing trend in the CD4^+^ T-cell counts and a greater number of animal deaths [16]. However, SFV pVLs were not assessed in that study. Herein, with the determination of pVLs of two feline retroviruses (FFV and FeLV), it was possible to observe that FeLV progressive or regressive infections were not able to reactivate FFV from latency in PBMC. However, a recent study showed that FFV pVL in blood was positive correlated in cats with status of FeLV progressive, presence of FeLV-B subtype and positive status for feline coronavirus (FeCoV), demonstrating a mechanism of complex interaction that need to be clarified [62]. Furthermore, co-infected cats with progressive FeLV infection are likely more prone to become FFV-transmissible and *vice-versa* as evidenced by the higher pVLs in these oral compartments. As the proportion of potential-transmissible FFV infections was similar between FFV monoinfections and in cats with progressive FeLV infections, but lower in regressive FeLV co-infections, we postulate that the latency mechanism in regressive FeLV could also contribute to reduction of the establishment of FFV colonization of the oral cavity. These findings may also be a consequence of an altered disease susceptibility caused by the cats’ virome composition. The virome influences the phenotype of the host in a combinatorial manner by interacting with other components of the microbiome and by interacting with individual variations in host genetics. Together these interactions may influence a range of phenotypes as we observed in the distinct behavior of FeLV progressive and regressive infections as also in the FeLV regressive correlation with reduced FFV saliva transmission [63]. The microbiome composition may also allow the stimulation of specific immune cell subsets, which may carry the FFV proviral DNA, thus indirectly increasing the FFV pVL due to the increased number of cells. We also found that FFV pVL was higher in buccal tissues of FFV/FeLV co-infected cats than among FFV monoinfections. However, we identified a negative correlation between the pVLs of both viruses circulating in this tissue. A previous study has reported FeLV as a cofactor for FIV infection by enhancing the expression and spread of FIV in the host and by increasing the severity of immunodeficiency caused by FIV by an unknown mechanism [42]. Another study evaluated FIV/FeLV interactions in in vitro and in vivo experiments, but did not find evidence of direct viral interactions [63]. Since FeLV is mainly transmitted through saliva [30] and superficial differentiated epithelial cells of the oral mucosa are the major cell types in which FFV replicates [51], we hypothesize that both viruses can infect the same compartment. Just like the Tax protein of HTLV-1 can activate HIV expression in vitro via the LTR [64], FeLV may enhance FFV replication in the oral cavity by a similar mechanism, or related to regulatory protein synergism, or some other means. A simplified model of this hypothesis is presented in Figure 6. Any potential interaction mechanism between these two viruses, and their infectiousness, remains to be determined and any contributions of infectious DNA-containing viral particles compared to integrated proviral DNA sequences was not measured in our study due to the small volumes of materials obtained for analysis.

Although we found evidence for increased buccal FFV pVLs in cats co-infected with FeLV with the potential for increased transmissibility, we did not observe significant disease associations in these cats. A small number of dually infected cats with FeLV-progressive infection had anemia but this trend was not highly significant. We also did not find large numbers of FIV-infected cats to evaluate the effect of FV co-infection on disease outcomes. Additional studies with larger numbers of naturally or experimentally infected cats and longer periods of follow-up may be needed to determine these potential associations.

Our study also reinforces the importance of FeLV qPCR testing to define FeLV infection outcomes, enabling differential clinical treatment of cats that were negative by serological methods. For example, blood transfusion from regressive cats containing latent feline leukemia provirus caused infection and disease in naïve recipient cats [61]. Moreover, FeLV regressive cats with undetectable antigenemia, but that are immune to vaccination, are often equivocally vaccinated [59].

In summary, we provide evidence that FVs may interact with other retroviruses infecting cats. Our study supports that domestic cats naturally infected by FFV and FeLV can be used as a model to investigate the potential impact of FV on immunocompromised mammalian hosts. The results of these animal model studies may help inform the design of clinical and research investigations into the potential for SFV to cause disease in infected humans. The tools developed in our study will be useful to further explore consequences and interactions of multiple feline retrovirus infections of cats.

## Figures and Tables

**Table 1:** Primers used for PCR amplification of feline retrovirus (FFV, FIV and FeLV) genome sequences.

Virus	Genomic Region	Name	Sense	Primer Sequence (5′-3′)	Reference
FFV	LTR	FFV LTR out	1st forward	TGCACAGGAAGCTCCTTTAGGGTA	Designed herein
		789(rev)	1st and 2nd reverse	TCCCCACGTGTAGAGAAACACCACTC	[43]
		788(fow)	2nd forward	TGTACGGGAGCTCTTCTCACAGACTTGGC	[43]
	glycoprotein/	FFV_F1	1st forward	CCTCGGGTCCAAACACAGCC	Designed herein
	polymerase	FFV_R1	1st reverse	CCAAGGTACATCTCCAGG	Designed herein
	(*gag/pol*)	FUV2610s	2nd forward	AACAGCAACACTCTGATGTTCCCG	[44]
		FUV3107a	2nd reverse	ATATACATCTCCTTCCTGCGTTCC	[44]
	integrase (*int*)	SIF5N_mod ^a^	1st forward	TACATGGTTATACCCCACKAAGGCTC	[10]
		SIR1NN	1st and 2nd reverse	GTTTTATYTCCYTGTTTTTCCTYTCCA	[10]
		SIP4N	2nd forward	TGCATTCCGATCAAGGATCAGCATT	[10]
	envelope	env-f2	1st forward	GCTACTTCTACTAGAATAATGTTTTGGATA	[45]
		env-r2	1st reverse	AGCCACAGTAGTAATTGCATTGGCCAGGCC	[45]
		env-f3	2nd forward	GCTTTCAAAAATATGGACATTGTTATGTTA	[45]
		env-r3	2nd reverse	GTTTCTCCAAAATCTGCAAGCATATGGATG	[45]
FIV	reverse	RT out F	1st forward	GGAGTAGGAGGAGGAAAAAGAGGAAC	[46]
	transcriptase (RT)	RT out R	1st reverse	GCCCATCCACTTATATGGGGGC	[46]
		RT int F	2nd forward	GGGCCTCAGGTAAAACAGTGGC	[46]
		RT int R	2nd reverse	GTCTTCCGGGGTTTCAAATCCCCAC	[46]
FeLV	envelope	Env Fow (1689 bp)	Forward	TCTATGTTAGGAACCTTAACCGATG	[47]
		Env Rev (1689 bp)	Reverse	CAGAATATCTGTGGTAC AAGCCTTAA	[47]
		Env Fow (437 bp)	Forward sequencing	GCYTGGTGGGTCTTAGGAA	[47]
		Env Rev (437 bp)	Reverse sequencing	AACARAAGTAAAGACTGTTGG	[47]

^a^ The last four bases were removed from the original primer sequence (TACATGGTTATACCCCACKAAGGCTCCTCC).

**Table 2:** Socio-demographic data and FFV and FeLV prevalence ^a^ of domestic cats from Brazil in the present study.

Category	Number (%)	FFV Mono-Infected (%)	FeLV Mono-Infected (%)	FFV/FeLV Co-Infected (%)
Total	81 (100)	26 (32)	8 (10)	38 (47)
Gender (*n* = 81)				
Female	40 (49)	11 (28)	3 (8)	22 (55)
Male	41 (51)	15 (37)	5 (12)	16 (39)
Age group (*n* = 79)				
Kitten	18 (23)	8 (44)	0 (0)	7 (38)
Young	16 (20)	5 (31)	0 (0)	8 (50)
Adult	45 (57)	13 (29)	7 (16)	22 (49)
Neutered status (*n* = 66)				
Neutered/spayed	35 (53)	8 (23)	3 (8)	20 (57)
Intact	31 (47)	13 (42)	3 (10)	11 (31)
Household status (*n* = 78)				
Single	2 (2.6)	1 (50)	0 (0)	1 (50)
Multi-cat	21 (27)	2 (10)	3 (14)	11 (52)
Shelter	12 (15)	1 (8)	2 (17)	9 (75)
Feral	43 (55)	21 (49)	2 (5)	16 (37)
Outdoor Access (*n* = 29)				
Yes	9 (31)	0 (0)	3 (33)	5 (56)
No	20 (69)	4 (20)	1 (5)	12 (60)
Health status (*n* = 81)				
Healthy	27 (34)	8 (30)	2 (7)	11 (41)
Sick	52 (66)	18 (35)	5 (10)	26 (50)

^a^ Prevalence was calculated with number of infected individuals per total number of animals based on nested PCR/real-time PCR (FFV) or on serology/nested PCR/real-time PCR (FeLV) results. FFV, Feline Foamy Virus; FeLV, Feline Leukemia Virus. Feline immunodeficiency virus (FIV)-infected cats were not reported because only three FIV-positive cats were found (one coinfected with FFV and two multiply infected with FeLV and FFV).

**Table 3:** Detection of feline leukemia virus (FeLV) and feline immunodeficiency virus (FIV) in household and feral cats.

Virus	Category	Household (*n* = 35)	Feral (*n* = 43)	Total
**FeLV**	Seropositive only	3/35 (9%)	2/43 (5%)	5/81* (6%)
	PCR-positive only	4/35 (11%)	1/43 (2%)	5/81* (6%)
	PCR-negative and seropositive	9/35 (26%)	8/43 (19%)	17/81* (21%)
	PCR-negative and seronegative	19/35 (54%)	32/43 (74%)	51/81*(63%)
	Total FeLV infections	16/35 (46%)	11/43 (26%)	27/81* (33%)
**FIV**	PCR-positive	0/35	1/43 (2%)	1/81* (1%)
	Seropositive	1/35 (3%)	1/43 (2%)	2/81* (2%)
	Total FIV infections	1/35 (3%)	2/43 (4.7%)	3/81* (3.7%)

* The total number includes 35 household cats, 43 feral cats and three cats with no information.

**Table 4:** Nested FFV PCR detection in peripheral blood mononuclear cells (PBMC) and buccal swabs.

Fragment	PBMC (%)	Buccal (%)
long terminal repeat (LTR)	15/79 ^b^ (19)	0/67 ^b^ (0)
*gag*/polymerase (*pol*)	27/81 (33)	13/68 (19)
Integrase	14/81 (17)	9/68 (13)
Envelope	19/81 (23)	1/43 ^b^ (2)
Nested FFV PCR-positive cats ^a^	33/81 (41)	19/68 (28)
FFV qPCR-positive cats	53/73 (73)	19/64 (30)
Total FFV-positive cats	62/81 (77)	31/68 (46)

^a^ Cats with at least one virus fragment detected. ^b^ In three cases, a limited number of samples were tested due to material availability.

**Table 5:** Univariate analysis of the prevalence and risk factors for FeLV and FFV co-infection in domestic cats.

Factor	Category	FeLV Co-Infection Prevalence	Odds Ratio	95%CI	*p* Value
Sex	Female	0.67 (22/33)	1.87	0.683–5.148	0.223
	Male	0.52 (16/31)	Reference	-	-
Age	Adult	0.63 (22/35)	1.47	0.534–4.03	0.458
	Non-adult	0.53 (15/28)	Reference	-	-
	Young	0.61 (8/13)	1.1586	0.332–4.046	0.817
	Non-young	0.58 (29/50)	Reference	-	-
	Kitten	0.47 (7/15)	0.5250	0.1628–1.6927	0.281
	Non-kitten	0.62 (30/48)	Reference	-	-
Neuter Status	Neutered	0.71 (20/28)	2.954	0.9378–9.3088	0.064
	Intact	0.46 (11/24)	Reference		
Household	Multicat	0.73 (11/15)	2.221	0.6171–7.9947	0.222
	Non-multicat	0.55 (26/47)	Reference	-	-
	Shelter	1 (9/9)	ND ^a^	ND	ND
	Non-shelter	0.47 (28/53)	ND	ND	ND
	Street	0.43 (16/37)	Reference	-	-
	Non-street	0.84 (21/25)	6.89	1.971–24.088	0.0025
	Single	1 (1/1)	ND	ND	ND
	Non-single	0.59 (36/61)	ND	ND	ND

^a^ ND = not done. There were no individuals for all comparison groups.

**Table 6:** Proportion of major clinical symptoms by feline retrovirus infection status.

Infection Status (*n*)	Respiratory Tract Disease (%)	Progressive Weight Loss (%)	Ocular Secretion (%)	Anemia (%)
FFV-monoinfected (*n* = 26)	6 (23.1%)	4 (15.4%)	5 (19.2%)	0/1 (0%)
FeLV regressives (*n* = 23)	11 (47.8%)	6 (26.1%)	2 (8.7%)	0/2 (0%)
FeLV progressives (*n* = 19)	6 (31.6%)	5 (26.3%)	0 (0%)	6/8 (75%)
FFV negative/FeLV negative (*n* = 5)	1 (20%)	0 (0%)	0 (0%)	N/A ^a^

^a^ N/A = not available.
